# Third Party Certification of Agri-Food Supply Chain Using Smart Contracts and Blockchain Tokens

**DOI:** 10.3390/s21165307

**Published:** 2021-08-06

**Authors:** Ricardo Borges dos Santos, Nunzio Marco Torrisi, Rodrigo Palucci Pantoni

**Affiliations:** 1 Center of Mathematics, Computing and Cognition, Federal University of ABC, Campus Sao Bernardo do Campo, Sao Paulo 09606-070, Brazil; ricardo.borges@ufabc.edu.br (R.B.d.S.); nunzio.torrisi@ufabc.edu.br (N.M.T.); 2 Department of Eletrical Engineering and Computer Science, Federal Institute of Sao Paulo, Campus Sertaozinho, Sao Paulo 14169-263, Brazil

**Keywords:** blockchain, agri-food, supply chain, third party certification, green-washing, Ethereum tokens, ERC-20, ERC-1155, distributed ledger technology, non fungible tokens

## Abstract

Every consumer’s buying decision at the supermarket influences food brands to make first party claims of sustainability and socially responsible farming methods on their agro-product labels. Fine wines are often subject to counterfeit along the supply chain to the consumer. This paper presents a method for efficient unrestricted publicity to third party certification (TPC) of plant agricultural products, starting at harvest, using smart contracts and blockchain tokens. The method is capable of providing economic incentives to the actors along the supply chain. A proof-of-concept using a modified Ethereum IGR token set of smart contracts using the ERC-1155 standard NFTs was deployed on the Rinkeby test net and evaluated. The main findings include (a) allowing immediate access to TPC by the public for any desired authority by using token smart contracts. (b) Food safety can be enhanced through TPC visible to consumers through mobile application and blockchain technology, thus reducing counterfeiting and green washing. (c) The framework is structured and maintained because participants obtain economic incentives thus leveraging it´s practical usage. In summary, this implementation of TPC broadcasting through tokens can improve transparency and sustainable conscientious consumer behaviour, thus enabling a more trustworthy supply chain transparency.

## 1. Introduction

### 1.1. Sustainable Farming and Consumer Choice

Healthy food starts with honest farming and harvesting. Several factors will, however, influence the exact quality and properties of each instance of the food that reaches the consumer. Examples would be the farming methods, seeds, micro-climate, humidity during the harvesting days, or need to use some agro-chemical against weeds or insect infestation. The storage at the farm as well as the handling along the supply chain links can also mean that quality ingredients do not reach the consumer’s table.

In addition to the increasing pressure for greater productivity, farmers are being pushed by end consumers to use more environmentally friendly procedures and agricultural methods. Consumer awareness that their buying decision at each visit to the supermarket can positively influence social and environmental equilibrium moves brands to make claims of sustainable farming methods on their labels. This trend began with organic production and fair trade initiatives and has grown to a wide variety of certification methods.

Certifications are available for crops such as sustainable cocoa, pineapple, cattle, and palm oil. Certification strategies are gaining widespread customer support and constitute an important vector in changing how food will be obtained and distributed in the future.

In 1990, the Organic Foods Production Act (OFPA) established standards for agricultural producers of commodities that claimed to use organic methods. The methods, practices, and substances used in agricultural practice, including sowing; growing; and harvesting; as well as handling crops, livestock, and processed agricultural products, restrict the wording on the product labels and marketing. Since OFPA, the US consumer has been continuously increasing demand for certified organic foods brands that claim to use organic production processes. Nevertheless, these organic farm certification methodologies have shown limitations and criticism: the authors of [1] conclude that the “current regulatory framework is not only inadequate to the task of regulating domestic organics, but also incapable of ensuring the integrity of imported organics. Thus, the USDA Organic seal misleads consumers”.

The term green-washing was defined [2] as “communication that misleads people into forming overly positive beliefs about an organization’s environmental practices or products”. This procedure jeopardizes environmentally friendly consumer behaviour.

### 1.2. Need for Third Party Certification

Several studies have recently claimed that certification of products hold great beneficial potential, such that [3]: “Product certification is one of the most promising and developed instruments to reward the socially and environmentally friendly practices of market producers”.

Third party certification (TPC) differs from first and second party certification mainly because the third party authority that issues the certificate has no interest in the transaction. A TPC involves an “independent Organisation with expertise to provide an assessment and verification of the company’s compliance with standards or legal requirements” [4].

In the last few years, TPC has become a growing regulatory mechanism in the global plant, agricultural and food industries. This trend represents a major shift from public to private governance demands and methods. The rise of TPC in the agri-food sector will allow for more transparency and accountability of negligent or fraudulent market participants. This phenomenon is akin to the great advances in quality and transparency that the third party certification trend brought about by the ISO2001 certification of industries and services in the last decades.

TPC can be very useful to ascertain product physical, chemical, or organoleptic properties and is allowing bolder certification of social, environmental, and sustainability properties. According to the work in [5]: “TPC also offers opportunities to create alternative practices that are more socially and environmentally sustainable”.

### 1.3. Certification of Farms versus Certification of Each Harvest

Although the farming procedures may be certified according to criteria such as quality, sustainability, or social fairness, there is no form of ensuring that certification of the typical farming methods, such as USDA Organic certification methodology, avoids that specific harvests are stained by malpractices such as agrochemical exposure or used hidden child labor.

Each harvest of a specific crop is unique. The difference may lie in the seeds used for that particular season or in the total hours of sunshine in that specific location during the crop’s growth.

In several agricultural sectors, especially in the wine trade, consumers recognize the crop timing and the different characteristics between harvest of different years even from the same farm. The analysis of the organoleptic properties of the wine produced recognizes major differences in year and location of the harvest of grapes. In the wine sector, the wine counterfeit problem can be summarized as avoiding the problem where larger quantities of more valuable wine from grapes harvested on better years or regions reaches retail than the volume actually produced. This fraud is also known as the mass balance problem [6] or double spending [7] and is very deleterious to the business as it can stain the reputation of premium producers. Products that are geographically traceable to a specific region, i.e., reserved by local laws under the protected designation of origin (PDO) concept also frequently suffer from this type of fraud.

A harvest TPC mechanism with tamper resistant certificates which are easily available to any stakeholder via mobile devices is needed to avoid double spending and significantly boost trust along the supply chain.

Thus, the following main research questions (RQ1) and subsidiary research questions (SRQ2, SRQ3) are posed:

**RQ1:** “Is it possible to establish a harvest TPC mechanism with tamper resistant certificates easily available to anyone, even previously unknown food supply chain stakeholder via mobile devices?”

**SRQ2:** “If one such mechanism is possible, who will carry the data input and maintenance costs? In other words, how will each stakeholder be incentivized to use this mechanism?”

**SRQ3:** “If one such mechanism is possible, what would be the typical time for the response to a certification query, in other words what quality of service can be expected by the end consumer?”

### 1.4. Proposed Solution and Structure of This Document

We propose to use Ethereum-based tokens and smart contracts pointing to TPC certificates for keeping track of certificates for individual harvests of each farm. In this manner, we show that it is possible to track the exact origin and quantity of each harvest from farm to consumer, offering the benefits of TPC available to the last links of the chain. Practical economic incentives to the chain participants are described allowing for effective productive usage. The focus is on information availability, reliability, synchronization to the physical flow of goods, and, above all, ensuring good publicity of the certificate at the consumer level.

This research paper is structured as follows. Section 2 presents relevant concepts and literature of food traceability, blockchain, smart contracts, and distributed ledger technology (DLT) and the Ethereum-based non-fungible token (NFT). Section 3 discusses the requirements and implementation of a token passing TPC framework using the ERC-1155 token smart contracts. Section 4 analyses the results obtained. Section 5 presents the conclusions pinpointing the research’s main contributions and limitations.

## 2. Related Work

### 2.1. Main Concepts in Traceability of Foods

Food traceability and food recall actions research has established important concepts. The Institute of Food Technologists [8] has been able to demonstrate that the sufficient and necessary conditions to maintain traceability of foods in the six families of foods are as follows:

- “Critical Tracking Event [8]” (CTE) (http://www.ift.org/gftc/~/media/GFTC/Events/BestPracticesinFoodTraceability.pdf accessed on 3 August 2021) defined CTE which is understood as a circumstance in which a traceable resource unit (TRU) undergoes change of ownership or(( custody or looses part or all its mass.
- “Key Data Element [8]” (KDE) (http://www.ift.org/gftc/~/media/GFTC/Events/BestPracticesinFoodTraceability.pdf accessed on 3 August 2021) defined KDE as the information that needs be stored for full provenance, at each CTE, i.e., to characterize the change of custody or loss of part or all of the mass.

The work in [9] provides a comprehensive overview on the application of blockchain technology to agri-food value chains. These are in line with the work in [10] which concludes that the use of blockchain technology can improve sustainability from social, environmental, and market perspective. In [11,12,13,14], blockchain tools are referred to as possibly the most appropriate to meet the requirements of the rapidly expanding food value chains such as traceability, auditability, fault tolerance, and flexibility. Research on certification using blockchain [15] has also evolved with many interesting sustainability efforts.

### 2.2. Blockchain

Bitcoin [16], the first successful cryptocurrency, was capable of validating the concept of a scarce digital object. The blockchain replicates an unknown number of copies of consistent data. A chain of transactions, organized into cryptographically linked blocks, could, for the first time, reach a consensus, even within a (limited) number of unreliable (traitor) nodes. For a more detailed description of the data structures involved see in [17,18]. Albeit the eventually synchronized nature of the protocol and possible temporary partitions in the network, the linear block of data is re-established after a partition, and regains consistency and availability.

The protocols behind Bitcoin, namely, the blockchain consistency mechanism, is maintained by worker nodes. These nodes are referred to as miners because they receive rewards in the form of Bitcoins to find and keep a distributed consensus among the various persistent data sources. Consistency of distributed data within a predetermined time frame is achieved, avoiding the double spending [7] of the digital asset.

The technology behind blockchain successfully implements consistency and access discipline for collaborative data in a diffuse globally distributed accessible trustless environment. The consistency achieved by the underlying data structures and control mechanisms with validation through the consensus of third party validators or miners shows that this technology is an important step towards supply chain transparency and traceability data [19,20,21].

A blockchain is a cryptographically auditable, append-only, tamper-resistant, distributed and replicated data structure, accessible to anyone employing a web browser. Blockchain can store structured data as well as methods or programs to process this data according to deterministic program steps known as smart contracts. Blockchains require no central trust mechanism, thus there exists no central point of failure. The main strengths of Blockchain Technology (BCT) are listed below.

- Tamper resistance, i.e., cryptographic hashes to previous block, in practice, make it impossible to change data that has been recorded;
- Pseudo-anonymity, i.e., data are available publicly but encoded through hashed keys that allow for trust on the existence and on the authorship;
- Distributed presence, i.e., the data structures are replicated maintaining several copies with no single point of failure and keeping integrity between data sets;
- Software-driven, i.e., the Blockchain mechanism does not require human privileged operators to maintain the transactions, thus the system is not prone to bribery;
- Allows for certification of the tamper-proof storage of off-chain data by means of side blockchain. These are hierarchically hash certified sub-database which can store larger volumes of data, including multimedia and providing evidence and tools for more detailed analysis.

The main BCT limitations today are very high energy consumption for the blockchains using the Proof-of-Work (PoW) consensus [22] and slow confirmation of transactions, typically 10 to 20 min for bitcoin and difficulties in scalability. All of the above limitations are actively being investigated by many practitioners and researchers today with very positive perspectives.

### 2.3. Smart Contracts, Distributed Ledger Technology (DLT), and Tokens

Ethereum [23] expanded the concept of the blockchain to distributed ledger technology by including tokens and programs called smart contracts that are executed independently of human intervention. These are open-source, human-readable high level programs that are stored on the blockchain and run inevitably, without any human intervention, strictly as implemented thus avoiding any risk of downtime, censorship, or fraud [24]. The Ethereum Virtual Machine implements “unstoppable” and “unavoidable” Turing-complete computer processes. Smart contracts use open-source code and are developed to establish standard behaviour between blockchain stakeholders and other contracts. They allow for extensive development and precise control, ensuring transparency of each data manipulation and thus trust.

Tokens are digital objects capable of representing object properties, assets, or rights that have strict transactional behaviour and ownership. The execution of the smart contracts is immune to any human interference and therefore allows for transparent systematic transactions. Tokens can be used to represent supply chain, intellectual properties, voting, or identity management systems, among other objects. The associated smart contracts assure discipline to the corresponding state transitions of token balances and thus generate trust to the parties without a trusted third party or a single point of failure. This assures transparency and prevents possible “double-spending” frauds in certification system.

### 2.4. Blockchain Architectures: Public vs. Private Read, Permissionless vs. Permissioned Write and Validate

The access permissions to reading, writing, and validating blocks on a blockchain system follow different models. It is necessary to distinguish between public blockchains, which allow universal read operations, from private blockchains, which allow read operations only by predefined users. Further, for operations such as write and commit (validate) blocks, two types of blockchains models are possible: permissionless blockchains allow universal write and commit commands as opposed to permissioned blockchains, in which only pre-qualified users are allowed to issue write or commit commands. Table 1 summarizes these architectures.

### 2.5. Digital Tokens Families

**Figure 1:**
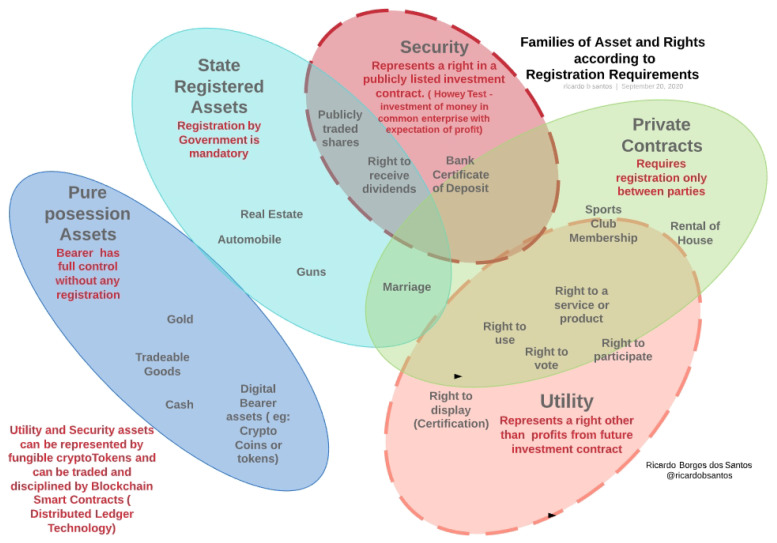
Families of assets and rights according to registration requirements (source: the author).

Tokens are digital objects that represent specific rights or assets. They should be understood as assets that can be negotiated or used as guarantees. Note that the necessary and sufficient condition for full ownership of the balance of the token on a public address is the knowledge of its private key. Figure 1 shows an Euler–Venn diagram for most common assets and rights, grouped into families along with their corresponding registration requirements.

The registration of the rights and property of assets, if required by law or regulation, will usually be centralized at a government-trusted centralized database. Because these data are maintained in centralized databases they are prone to corruption, fraud, censorship, downtime, or misuse. On the other hand, distributed registration schemes based on replicated databases, such as distributed ledgers, provide very high availability, are fraud-resistant, are fault-tolerant, and typically cannot be censored. Security and utility assets can reliably be represented, registered, and easily traded as cryptographic tokens. Automated processes through smart contracts allow high availability, low costs of transaction, full traceability, non-repudiation, and pseudo-anonymity.

In order to be useful, tokens should not be copyable (i.e., should not be prone to double spending attacks) or suffer arbitrary changes. Thus, they need to follow a strict discipline at each change of state to usefully represent real world objects.

The development of digital objects to simulate real-world objects requires that the objects properties and behaviour are modeled through common data structures and coded procedure. Smart contracts manipulating tokens must respect some standard to allow for multiple users and contracts to share functionalities among different applications. Application independency and fungibility of digital objects could be achieved with a minimum set of functionalities. The ERC-20 token fungible objects standards are key to the success of many cryptocurrencies and many Ethereum decentralized applications. Because the ERC-20 token metadata structure holds all relevant property data within the blockchain, they can be freely transferred from one blockchain to another, allowing these to be exchanged for other ERC-20 assets.

It is important to note that alike a real estate property record, which entitles the bearer to have full use and ownership of a real estate asset, the possession of a private key of a token on one blockchain entitles that person or smart contract to unrestricted use of that token for payment, exchange, deposit as warrant or collateral, lend or sell this assets at his discretion.

Further, it is important to recognize that objects can be categorized in fungible objects and non-fungible objects. **Fungible objects** are those that need not be distinguished from one another. The important question here is “How many of these objects?”. **Non-fungible objects**, on the other hand, are those that are distinguishable from similar objects. The decisive question here is “Which of these similar although unique objects?”

The distinctive property between fungible and non-fungible tokens is that the former are fully exchangeable and thus can be added, e.g., coins of same face type and value can be added or subtracted at will. The latter, not being exchangeable, can only be transactioned as unique identifiable objects.

A non-fungible token (NFT) is a unique blockchain-based digital entity which can represent a non-fungible object. If this token follows a protocol such as the ERC-1155 or ERC-721, it can be traded as an asset between various stakeholders in possibly multiple applications.

The methods defined in the ERC-1155 standard assure consistent behaviour, transparency, no double spending and a verifiable auditable trail to families of similar, yet unique, objects. An ERC-1155 compliant NFT has one identifier that points to a specific URI, in which typically all properties and details are described. Additionally a numerical characteristic of this object is also available (https://github.com/ethereum/EIPs/blob/master/EIPS/eip-1155.md accessed on 3 August 2021).

Table 2 summarizes and compares the applicability of each standard.

### 2.6. Applications of DLT to Supply Chain Management

DLT has the potential to monitor social and environmental responsibility, improve provenance information, facilitate mobile payments, reduce transaction fees including credit and financing operations, and manage supply chain in a secure and trustworthy way.

Recent research literature searches for keywords such as “supply chain”, “food traceability”, and “blockchain” covers several reviews pointing to many interesting use cases. Benefits, disadvantages, and trade-offs of using blockchain technology to promote trust among the supply chain participants for food traceability have been discussed in detail [9,10,25,26,27,28,29,30,31]. However, none of these references addresses reliable third party certification information flow from farm to consumer.

Another important initiative is TE-FOOD (https://tefoodint.com/ accessed on 3 August 2021), a Hyperledger-based traceability product largely used to trace meat, vegetables, and produce. Whereas TE-FOOD has been commercially very successful, the TE-FOOD methodology today is unable to trace or certify commingled products such as the “bakery”, “processed food”, or “dairy” products, as described in [8]. Further, by allowing the farmer to directly register instances of his harvested products, it is basically a first-person certification scheme, lacking the necessary credibility for end consumer.

### 2.7. Consumer Valued Properties Tokens in Agro Products

For decades, important crops have been traded as commodities. Commodities are intrinsically fungible. Once the product is classified in a certain grade, according to purity, size, or maximum cross-contamination levels, then the lot is handled as a commodity. Global trading standards and procedures require that a bushel of wheat is fully fungible with another bushel of wheat.

However, a specific harvest is a unique object. No other harvest possesses the exact same properties, therefore harvests are non-fungible physical objects. To track this object appropriately, it is necessary to record all relevant data which will individualize and keep the history of that specific harvest product.

Food consumers are becoming more demanding and requiring different processes and raw-materials. Claims such as “non-genetic-modified seeds”, “organic crop cultures”, “agro-chemical-free”, and “gluten-free” are more present in food labels and adverts than ever. Nevertheless, food suppliers are seldom willing to provide evidence for those claims. In many cases the claims cannot be evidenced and must be understood as mere “green-washing”.

In [32], a DLT-based system is described to record a specific harvest from seed supplier to end customer using smart contracts. The mechanism allows for registering each step of the value chain, but does not allow for a third party (authority) certification, and therefore is more prone to fraud. Further, the tiresome recording of each link in the chain would be relegated without some type of incentive.

In [33], an ERC-20 Ethereum token, named the IGR token, and its smart contracts is shown to allow consumers to evidence TPC for any “consumer value perceived property”. This includes properties in physical, biological, social, or environmental domains. The framework was developed for TPC of ingredients of commingled products. The major concern was to retain traceability and certification without requiring brand owners of these commingled products to jeopardize their trade secrets such as recipes or ingredient supplier base. The IGR token allowed for a higher level of consumer trust in the commingler’s claims on their labels. The framework is applicable to a large spectrum of foods such as prepared foods, baked goods and commingled foods. For the other food families, such as produce, meat and poultry and seafood the traced item usually follows the supply chain links as a single whole item, or in parts, but is never mixed to other “ingredients”.

### 2.8. Why Non-Fungible Tokens

The original IGR token framework does not differentiate between tokens obtained from different harvests because it considers the tokens to be fully interchangeable, i.e., fungible. This is not an accurate picture of the real world and would allow potential fraudulent usage of tokens. For example, a malicious user of the IGR token could acquire tokens of the same value property description but from different harvests and use all tokens as if they were of the more valuable source. In other words, although different harvests have different perceived value, the original IGR smart contract implementation allowed malicious users to use any tokens for the same property as if all were from a more valuable harvest.

We made changes to the original IGR smart contracts to avoid this type of attack and to ascertain that any IGR-token certified object or harvest is handled as a non fungible object.

## 3. Research Methodology

**Figure 2:**
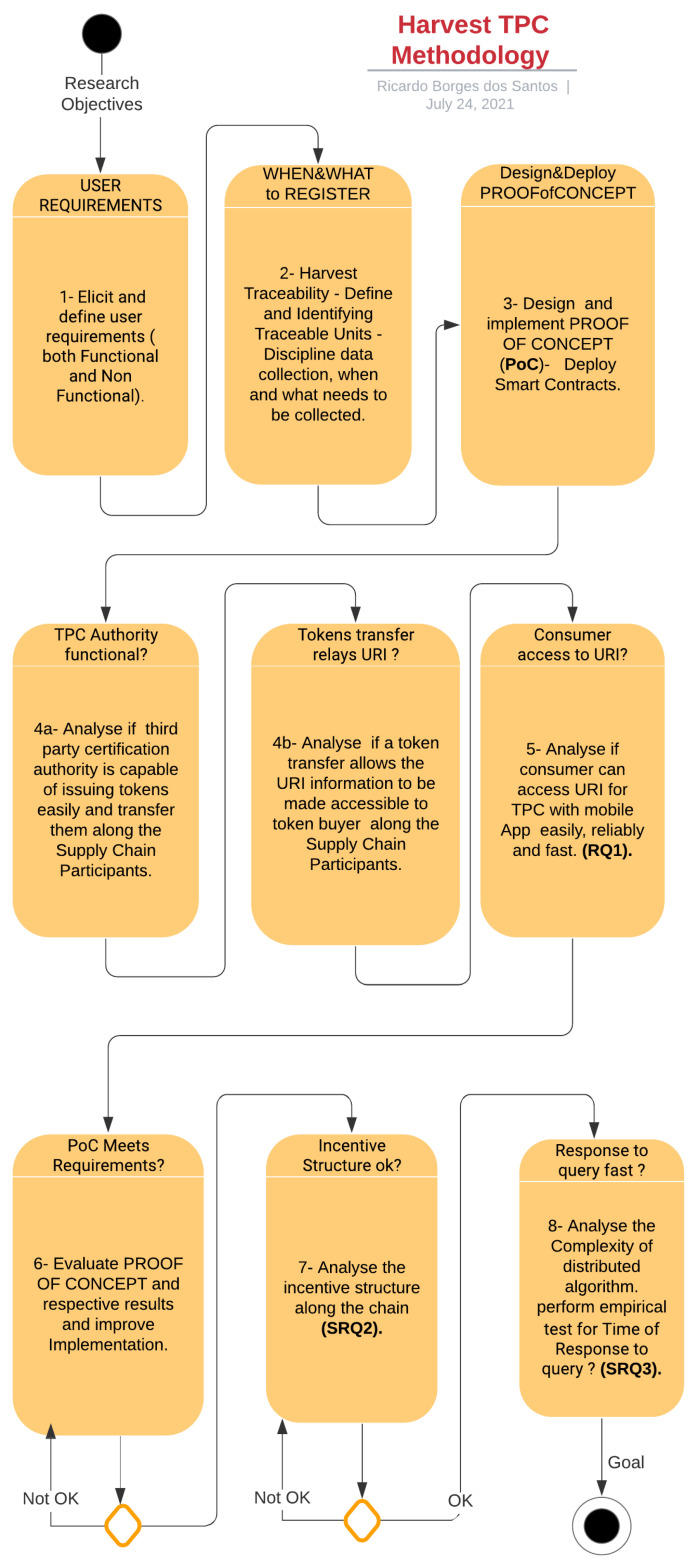
Methodology for Harvest TPC validation using Proof-Of-Concept Smart Contracts (source: the author).

The research methodology chosen to systematically answer the Research questions RQ1 and subsidiary research question SRQ2 and SRQ3 involves designing a proof-of-concept set of smart contracts, then deploying them and testing for the design requirements, and finally evaluating the research objectives and questions. The detailed procedure is depicted in Figure 2. The diagram shows a step by step description of the methodology for Harvest TPC validation using a Proof-Of-Concept (PoC) set of smart contracts.

In summary, the methodology systematic develops the following steps:

- 1—Elicit and define user requirements (both Functional and Non Functional).
- 2—Harvest Traceability - Define and Identify Traceable Units - Discipline data collection, i.e., when and what needs to be collected.
- 3—Design and implement proof-of-concept (PoC)—Deploy smart contracts.
- 4a—Analyze if third party certification authority is capable of issuing tokens easily and transfer them along the Supply Chain Participants.
- 4b—Analyze if a token transfer allows the URI information to be made accessible to token buyer along the Supply Chain Participants.
- 5—Analyze if consumer can access URI for TPC with mobile App easily, reliably, and fast (RQ1).
- 6—Evaluate PROOF OF CONCEPT and respective results and improve Implementation.
- 7—Analyze the incentive structure along the chain (SRQ2).
- 8—Analyze the Complexity of distributed algorithm. Perform empirical test for Time of Response to query? (SRQ3).

Note that the very essence of the blockchain immutability helps any researcher to trace all the test runs and deployments of the smart contracts by means of any blockchain scanner. This allows the research methodology and procedures to be easily reproducible and traceable (Examples of blockchain scanners are https://www.etherchain.org/, https://www.EthPlorer.io or https://www.Etherscan.io all accessed on 3 August 2021). In other words, both the smart contract source code as well as all the test runs of all test performed to the PoC can be followed in detail on any browser.

## 4. Proposed Architecture

### 4.1. Requirement Analysis

The desired functionalities of the system, i.e., the functional requirements are listed below.

- to allow for farmers to request any third person authority to inspect and certify properties that a specific harvest may have;
- to allow the inspection authority to issue a certificate in any web site including quantitative data about the desired property of the yield;
- to allow the authority to create (“mint”) tokens, i.e., digital objects representing the harvest and carrying the URI linked to the certificate, representing information about the mass of product inspected (yield);
- to allow these tokens to be “passed on” along the chain of buyers of the yield;
- to allow the buyer that applies the package, wrapper or label to the food product to write the URI to an easily and freely accessible reliable database and
- to destroy (“burn”) these tokens to avoid garbage or misusage, after a predetermined time.

As for the nonfunctional requirements, it is important to ascertain that the system brings the following:

(a) Universal access: allowing any supply chain participant, even previously unknown, to use the tool, without previous registration;
(b) Robustness to faults: allowing writing to a common persistent information layer in a robust manner;
(c) Cost effectiveness: allowing information to be recorded in an inexpensive manner;
(d) Tamper free auditability: enforcing tamper free, auditable transactions between any parties;
(e) No double spending fraud: avoiding that token balances are used more than once;
(f) Universal read access: allowing any potential consumer to freely read the certificate by means of a mobile device
(g) Usability: allowing for comfortable user experience.
(h) Quality of Service: guaranteeing that responses to a consumer query returns to the requesting device within short time period;
(i) Interoperability: allowing usage with different systems and devices and
(j) Scalability: allowing for a much larger number of transactions running within acceptable quality of service i.e performance.

### 4.2. Persistence Layer Design Alternatives

Although the above requirements point to a blockchain architecture, we asked: *Is a blockchain-based architecture, as opposed to a conventional centralized server–client database, persistence architecture really needed?*

If harvests are to be certified for the benefit of the entire chain of potentially stakeholders in the food industry, which type of data structure would be required to keep this information useful and trustworthy? In other words, is it necessary to use a blockchain to record and make all relevant information consistently available to all stakeholders?

**Figure 3:**
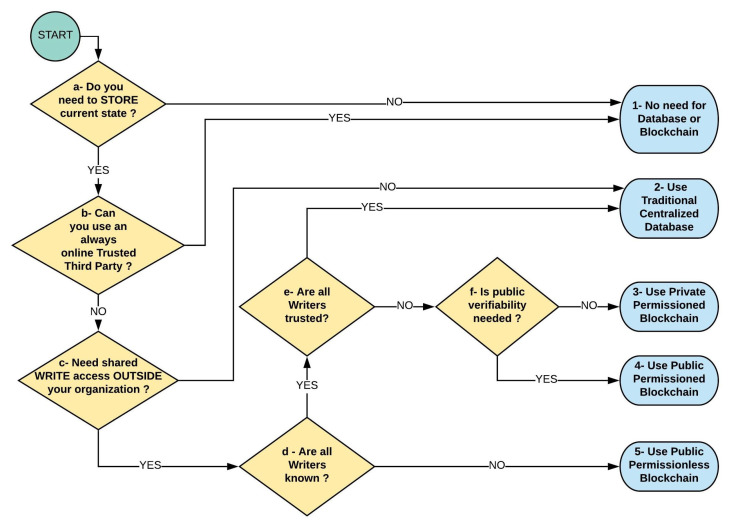
Procedure to define type of data persistence layer (database or blockchain) (source: adapted by author from the work in [35]).

Figure 3 adapted from in [34] shows a flowchart approach that helps analyze the data structure requirement for distributed systems. Note that our particular requirements for TPC of Harvest in the Food Supply Chain leads to the use of public permissionless blockchain as the best architecture. Applying the flowchart to the TPC of harvest we would ask:

- Is it necessary to store current State (Current Custodian on Supply Chain)? YES;
- Is a Trusted Third Party available online? NO;
- Is WRITE access needed outside your organization? YES; (because of the possibly many unknown chain participants).
- Are all Writers known? NO.

Thus, the recommended architecture is Public Permissionless Blockchain. Note that, because it is desired that the system maintains open doors to new entrants to the supply chain such as new farmers, known farmers with new crops, new mills, new re-sellers, new comminglers or new retailers, the choice of a permissioned blockchain such as Corda or Hyperledger was discarded.

As Ethereum meets all the non-functional requirements (a–i) listed above, the public Ethereum environment with the non-fungible token ERC-1155 standard protocol was chosen. The choice of using Ethereum may be considered suboptimal for the non functional requirement (j): scalability, but was chosen given the great advances this research theme is currently receiving from the academic and practitioner communities.

### 4.3. Implementation

The IGR token smart contract code used for ingredient certification in [33] was modified to implement the non-fungible token (NFT) discipline that better represent each instance of a crop with the use of the ERC-1155 (https://github.com/enjin/erc-1155 accessed on 3 August 2021) objects and methods. A set of public blockchain smart contracts govern the token synchronization framework to positively identify each harvest along the food supply chain to the end consumer. The fully documented source code for all the smart contracts in Solidity programming language was published in the Ethereum main net where all variables and algorithms are fully commented and documented. The code was developed, tested, deployed, and made available at https://etherscan.io/address/0x1448eab3182b71ae5322168d037feb0125cac92f#code (accessed on 3 August 2021).

**Figure 4:**
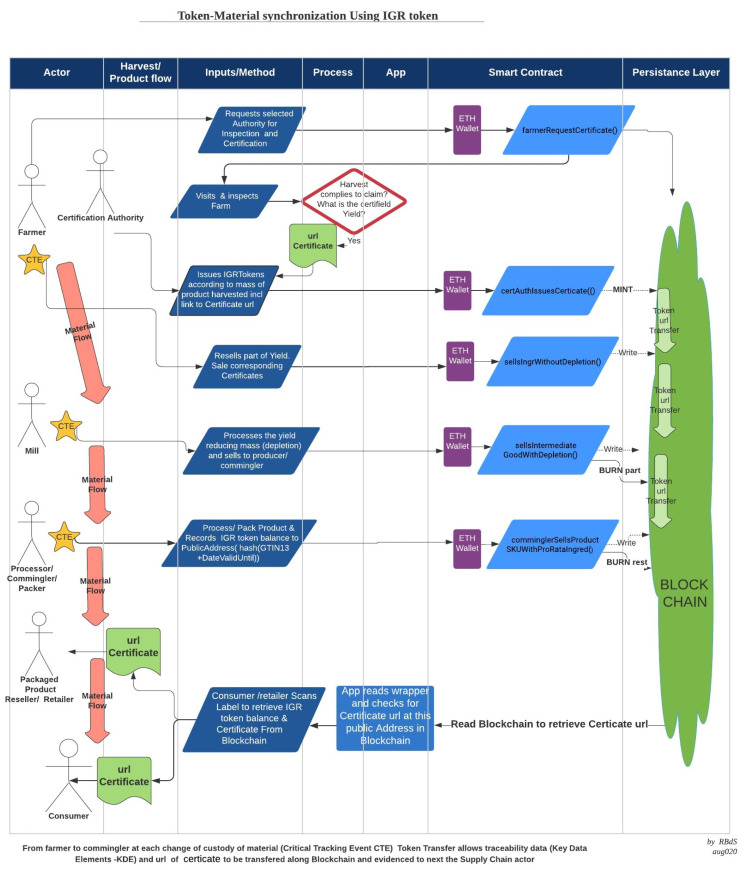
Synchronization of Food Lots of Harvests with Traceability Token (source: the author).

The token flow follows the commercial transactional changes of custody, usage, or depletion of the product. Figure 4 shows the synchronization between flow of food lots and the flow of certification data.

Using the IGR token set of smart contract after modifications to ERC-1155, the farmer responsible for the harvest can freely choose the properties to be certified between:

- functional (e.g., minimum size of fruit or grade);
- organoleptic (e.g., color or aroma);
- social (e.g., free of child labor cultures);
- environmental (e.g., “grown in certified no forest devastation areas”, “organic—no xyz herbicide”, or non Genetic Modified seeds only).

as well as the appropriate authority that will audit and issue the corresponding certificate for each harvest.

The authority is then invited to audit the farm at harvest time. After the appropriate auditing procedures, including inspection to the farm and qualitative and quantitative evaluation of crop yield, the authority formalizes the audit results by publishing the certificate as a webpage at the authorities domain web server.

The link to this certificate, in the form of the URI is part of the minting process. An example can be found at https://etherscan.io/tx/0x728ecf16e13939a62aba532b1df2f5b52ed9133ad0924c9109dc5b1fe5c6536f ( accessed on 3 August 2021). Further, this smart contract will issue the exact number of tokens to match the numerical mass yield of that specific harvest in grams. Thus, the ERC-1155 unified resource identifiers (URI) descriptor will point to the web page containing the full technical details of the certified “consumer valued properties”, including the original mass of goods in grams. The number of IGR tokens issued will represent this specific mass of ingredient.

**Figure 5:**
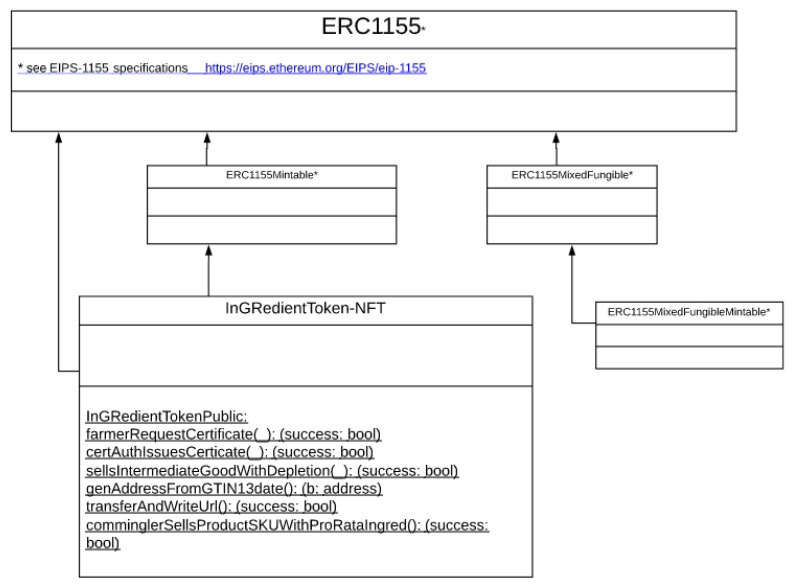
UML class diagram for the new implementation of the IGR Token (source: the author).

The smart contracts were coded in the high-level programming language Solidity. Figure 5 shows the simplified UML class diagram for the smart contract implementation (Details on ERC-1155 classes and methods available at https://eips.ethereum.org/EIPS/eip-1155 accessed on 3 August 2021).

Third party certification is assured. Note that, by using the delegated transfer “setApprovalForAll()” and “safeBatchTransferFrom()” primitive in the smart contracts, it is not possible for the farmer to issue or make first person claims on the certificate. Only the Authority has this capability, thus enforcing strict third person certification (TPC).

The ERC-1155 discipline allows for the farmer to sell all or part of the harvested product but stops any attempt to add tokens from different harvests. Just like in any blockchain, “double spending” of these tokens is not possible.

The necessary information in order to evidence to a final consumer that a specific harvest or food ingredient raw material was effectively inspected and certified by a third party to hold some “consumer value property” is handed over from chain participant to the next, all the way to the recipe final processor. The final processor, sometimes also known as commingler or packer, uses information printed on the product retail label to generate a Public key and link this to the certificate URI. Since most consumer products show a barcode (GTIN-13 SKU identifier) and a lot “consume before” date on the wrapper, these are used to provide an unique public key to the data stored in the blockchain. The hash of the “GTIN-13 + Date” string is the public key to the block chain. Querying the blockchain at this address reveals the certificate URI link. Access is facilitated through an App (available at https://play.google.com accessed on 3 August 2021) for Android mobile devices.

The farmer, based on his/her target consumer beliefs, defines a “consumer value property” and a certifying authority by using the smart contract *farmerRequestCertificate()*. After an inspection of the farm, the certification authority confirms the scope, quantity, and date of the lot produced and includes this information in the generated web page, with its URI under the authorityś web domain, certifying this information. This lot of crop will then be awarded an appropriate quantity of IGR tokens. For simplicity we define one IGR to be one gram of any certified ingredient, irrespective of the nature of the property. The authority issues IGR tokens through the smart contract *certAuthIssuesCert()* including nature, quantity, location and time of the harvest. Additionally the token holds the URI to the web page with all certificate details. Note that only the certification Authority has the permission to issue or not issue the tokens in the correct quantities. This assures the necessary independence of the third party certification and avoids conflicts of interest.

At each CTE where changes of ownership, custody or quantities of the raw-material are involved, a smart contract captures the transfer of custody data, such as mass reduction and writes these into the blockchain. These changes are automatically recorded by the sale of the tokens. The transactions are between pseudo-anonymous public Ethereum addresses, thus without disclosure of supplier identities or business critical data. At each transaction where the mass of the product is reduced the smart contract issues a proportional “burn” token command. At the packaging or commingler link, after the URI is written to the public address related to the hash of “SKU bar code + lot date” the rest of the tokens are “burned” to avoid misuse of any “zombie” tokens.

**Figure 6:**
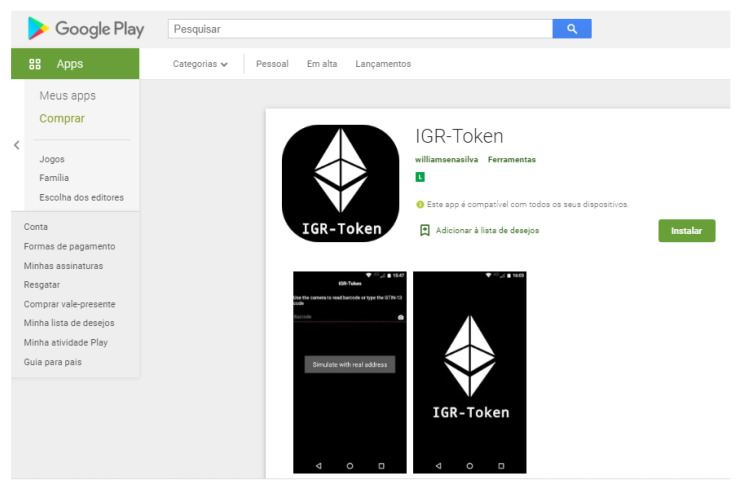
IGR Token Scanner and Certificate Look Up App on Google Play).

At the final links, a retailer or a consumer, by means of a mobile application, is able to scan the bar code on wrapper of the item, capturing the stock keeping unit (SKU) code and lot number to confirm the certification URIs. Figure 6 shows this App. The transfer of custody of the physical product and of the tokens are synchronized to the final consumer.

The tokens are unique and act as digital twins representing each quantifiable crop harvest instance. This allows TPC by authorities that will publish their specific audit report at their website of each harvest. The unified resource identifier (URI) of this certificate can be conveyed along the chain to consumers through a self sustained incentive mechanism.

The original IGR token smart contracts (ERC-20) was modified to use the ERC-1155 and deployed at the Rinkeby testnet. The Rinkeby test contract code at https://rinkeby.etherscan.io/address/0x841c5c79d9ae35db8fb4f216a478cd184fdae634#code (accessed 3 August 2021) was subjected to tests. The final code was deployed to the main net at https://etherscan.io/address/0xa63006f554566a788226d384bf70ed4e91851e05#code (accessed 3 August 2021).

Because of the blockchain immutability and traceability, all test runs leave a full trace-back log of events. These events can be reviewed on the Rinkeby or main net blockchains, as, for example, https://etherscan.io/address/0xa63006f554566a788226d384bf70ed4e91851e05#events (accessed 3 August 2021).

Testing, albeit limited so far, allowed to conclude that the code transfers the URI from original minter (authority) to each of following supply chain participants.

### 4.4. Incentives along the Chain

In order to cover the extra efforts with traceability documentation and data administration, the farmer and other stakeholders along the chain need some type of incentive. This is intrinsic to the framework as the farmer that decides to certify his or her product will be able to ask for higher sales price for his/her crop and transfer the tokens. Similarly, each participant in the custody chain can request a premium in price due to the fact that the merchandise is no longer a mere commodity. The end consumer will also pay a premium price for a certified product.

## 5. Discussion

In order to provide food value chain stakeholders an easily accessible, fraud-resistant tool to evidence that a specific harvest was environmentally and socially friendly, blockchain tokens and smart contracts are proposed as reliable certification tools.

Blockchain technology with appropriate smart contracts is proposed as a reliable methodology for certification of all types of harvest properties. Using this framework, farmers can be certified by any authorities and make their URI based certificates propagate along the supply chain links to the consumer. This provides food value chain stakeholders with a reliable tool capable of evidencing whether these harvests are environmentally and socially acceptable.

The proposed solution is helpful to avoid possible green washing attempts, thus preventing consumers being misled by unverified label information.

The use of distributed ledger technology (DLT) in the form of an Ethereum blockchain non-fungible utility token is shown to help fill this gap. The main advantages are low cost of transaction, providing incentives to each traceability partner along the chain and avoiding the mass balance fraud, also known as the “double spending” issue. This research implements a framework to support TPC on a harvest to harvest basis, as opposed to the farm processing method certification of, e.g., USDA organic farmer methods certification. Allowing farmers to select any certification authority enhances trust along the agro supply chain and empowers consumers to choose relevant certification and easily check for the TPC certificates directly via their mobile devices.

To meet the requirements of a third party certification scheme capable of certifying both physical properties, as well as social properties, the Ethereum IGR token and its set of smart contracts were modified to use the ERC-1155 discipline. A world wide web page URI with full details of the certificate is published by the authority, Thus, quantitative information on the yield is made available to the farmer to sell to the next supply chain participant in an incentivized structured manner. These tokens are passed along the chain and are capable of identifying a specific harvest and point to their unique third party certificates URIs (web link).

The Ethereum based IGR tokens and smart contracts for keeping track of each harvest of each farm offer the benefits of TPC. In this manner it is possible to track the exact origin and quantity of each partial element of the harvest from farm to consumer with TPC, ensuring proper economic incentives to the chain participants.

Suppose a bottler of wine intends to cheat and use a high priced harvest more than once (i.e., try to double spend the higher valued ingredient). We can show, by stepping through the smart contract code, that the IGR-1155 framework is capable of avoiding this attempted green-washing fraud.

**Figure 7:**
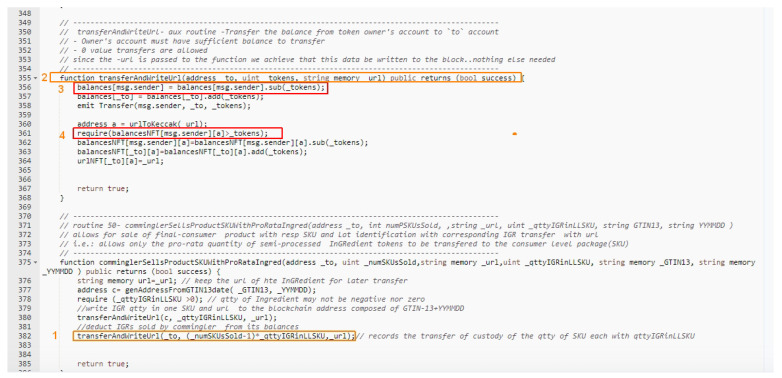
Smart contract snippet of the new IGR Token as deployed and in operation at the Rinkeby test network).

Figure 7 shows the smart contract code snippet for the method “Routine 50” and of its submethods.

If the commingler attempts to write to the Blockchain with sufficient IGR token balance of the same harvest, the code will be successfully executed and follow through the marked numbers 1–4. However, if the IGR token balance for the specific harvest is not sufficient, denoting a double spending attempt, the require() command will block the smart contracts from successfully executing at step 4 generating a fallback and therefore avoiding the fraud.

This paper significantly extended previous work [33], which focused on the traceability of commingled foods. The present research and smart contract code retains the original functionalities while extending the framework to allow for non fungible objects such as harvests of food products to be certified as unique objects. It has a major new focus on the conception, validation and usability of the smart contracts for TPC of non-fungible objects.

As answers to the main research questions **RQ1** and subsidiary Research question **SRQ2** and **RQ3** we conclude the following: 

**RQ1:** Yes, the modified ERC-1155 IGR token smart contracts publicly available on the Ethereum Blockchain are capable of demonstrating harvest TPC with tamper-resistant certificates easily available to anyone, even previously unknown food supply chain stakeholder via mobile devices, as shown by the proof of concept running on the Rinkeby test net described.

**SRQ2:** Yes, the mechanism establishes price incentives for each participant stakeholder. They will share the premium price the final consumer is willing to pay for the access to the TPC certification of products. The incentives at each link of the supply chain are limited to the premium the final link, i.e., the consumer actually pays. 

**SRQ3:** The typical time for the response for an end consumer to a certificate query using the Android App IGR-token described above is very comfortable because it uses only one direct hashed blockchain (linear data structure) access and one direct URI DNS web access to the certificate, both of which in linear time. This is due to the fact that, at each change of custody, the URI to the certificate is “handed over” to the next in the chain all the way to the commingler/packer. The public key information (GTIN-13 + lot date) to the certificate URI saved on the blockchain is on the product wrapper. 

In summary, the synchronization of the transfer of custody of the crop with the corresponding IGR token representing each gram of the yield instantiated for each different harvest is possible and incentivized using the modified IGR-token smart contracts suite. The modifications to the IGR-token code to use of the ERC-1155 disciplined methods maintains the previous functionalities enforcing necessary non fungible restrictions such that yields from different harvests now can not be added.

The most important limitation is that physical counterfeit of packaging, within a short period, i.e., re-utilization of original packaging material with counterfeit content whilst the spent tokens are still “live” can not be detected by the system. The methodology can not detect if packaging tampering shortly after the consumption may have led to “double spending” of the certificate.

Limitations to the current usage of the ERC-1155 IGR token framework may include the communication difficulty inherent to clearly defining and communicating the property being certified. In other words, the definition of the specific property, specially social or environmental, can be difficult to communicate: minor wording changes may cause consumer misunderstanding or wrong perception.

A further limitation may be the need, by the transacting parties in the supply chain, for some knowledge of token smart contracts or wallets and Ethereum usage. This requires development of user friendly front end applications. Note that the consumer does not require to write to the blockchain and can therefore directly obtain the certificate accessing the live Ethereum net (using a block scanner, e.g., www.etherscan.io) (accessed 3 August 2021) via the public key hash of the code plus lot date on the product wrapper.

Future work is required towards systematic testing of current IGR token smart contracts implementation, optimization of code to cater for “garbage collection” such as elimination of residual token balances, i.e., old token balances that may have been left after product being depleted as well as the development of optimized front-end apps with respect to user experience.

## 6. Conclusions

Farmers are under pressure to use more sustainable farming methodologies and simultaneously become more competitive. Some producers use labels inducing customers to believe that their ingredients are harvested in environmentally and socially friendly manners without proper evidence. Third party certification along with better availability of this information to the general public and supply chain actors can help fight this green-washing and promote consumer trust. Reliable publicity of the certificates with fast and easy access needs to be assured. Distributed ledger technology using tokens pointing to a certificate at the authority’s website, being transferred at each change of custody of the harvested crop along the chain, poses a practical solution. Transferring tokens with an embedded link to the certificate alongside the transfer of product at each of the transactions along the chain, provides traceability and integrates practical economic incentives. Non fungible tokens representing one harvest cannot be added to tokens representing another harvest. This behaviour is consistent with current commercial needs and practices. The Ethereum IGR token smart contracts were modified to use ERC-1155 non fungible tokens and thus enable tamper free TPC on a harvest to harvest basis, as opposed to farm certifications, such as Fairtrade or USDA Organic farm process certification. Main advantages of the framework are low cost of transactions, the incentives provided to each traceability partner along the chain, the disciplined mass balance, i.e., no “double spending” and the immediate access to the certificate URI through an internet portable device app.

The framework sets forth powerful incentives for the transacting parties to maintain a systematic recording of the required traceability data at each change of custody of the product. This is achieved by the economic incentive of the token transfer, synchronizing the appropriate amount of tokens with the transfer of custody of the product along the supply chain. The precondition for this intrinsic economical incentive is that the consumer, at the point of sale, is willing to pay a bonus for being able to readily view the TPC certificate. To do this, the consumer can scan the wrapper of the food item with a free Android App, and obtain the public key to the blockchain address where the URI of the TPC certificate is stored. The IGR smart contracts were rewritten using the ERC-1155 NFT methods and deployed. This proof-of-concept along with a front end Android app allow consumers to retrieve the TPC of the raw material at the retailer. The analysis of these distributed methods showed that the certificate’s URI can be retrieved in linear time, i.e., requiring one single access to the blockchain and to the corresponding TPC web page.

The main contribution of this research is to allow for effective TPC harvest instance certification, as opposed to more generic farm certification. It shows that true TPC, via the certificate URI at the authority’s website, can easily and publicly be made available via a mobile application. Note that the authority can be freely chosen by the farmer to allow for credibility among the target consumer. The authority certification discretion to certify or deny certification is fully protected, i.e., authorities are free to decide. The architecture privileges practical usage through inherent economic incentive to each stakeholder along the typical agro-supply chain links. This methodology enhances trust along the supply chain, allowing prompt and economical TPC publicity via mobile phones thus inhibiting “green-washing” attempts.

## Figures and Tables

**Table 1:** Types of blockchain architectures (source: the author).

	Public Permissionless Blockchain	Public Permissioned Blockchain	Private Permissioned Blockchain
**Read access**	Universal	Universal	Only pre qualified READERS
**Write access**	Universal	Only pre-qualified WRITERS	Only pre qualified WRITERS
**Commit access**	Universal (any node can be a VALIDATOR (miner)	Only pre-qualified VALIDATORS	Only pre qualified VALIDATORS
**Permissioned (Identity of Participants)**	No requirement	Enforces known and permanent Id for each participant	Enforces known and permanent Id for each participant
**Example**	Ethereum	Corda (https://www.r3.com/blog/how-public-permissioned-blockchains-are-not-an-oxymoron-2/ accessed on 3 August 2021)	Hyperledger Fabric (https://hyperledger-fabric.readthedocs.io/en/release-2.2/blockchain.html accessed on 3 August 2021)

**Table 2:** Comparison of Ethereum Request for Comments (ERC) tokens (source: the author).

	ERC-20	ERC-721	ERC-1155
**Nature of token**	Fungible	Non Fungible	Fungible/Non Fungible
**Limitation**	Each smart contract creates and handles one fungible token	One smart contract creates NFT only	One contract can create and handles Fungible and NFT
**Costs of transaction**	Cheaper	Expensive	Cheaper
**Efficiency**	High (fungible tokens only)	High (one of-a-kind token only)	High (fungible tokens and sets of NFT)
**Example**	EOS	CryptoKittens	Enjin

## Data Availability

Not applicable.
